# Tuberculosis diagnostic technology: an African solution … think rats

**DOI:** 10.4102/ajlm.v6i2.420

**Published:** 2017-03-31

**Authors:** Christiaan Mulder, Georgies Mgode, Stewart E. Reid

**Affiliations:** 1 Tuberculosis Department, Anti-Persoonsmijnen Ontmijnende Product Ontwikkeling (APOPO), Sokoine University of Agriculture, Morogoro, Tanzania; 2 Amsterdam Institute for Global Health and Development, Amsterdam, the Netherlands; 3 Centre for Infectious Disease Research in Zambia, Lusaka, Zambia; 4 Division of Infectious Diseases, University of Alabama at Birmingham, Birmingham, Alabama, United States

## Introduction

Tuberculosis has now gained ranking alongside HIV as one of the two leading causes of death from infectious diseases worldwide.^1^ In 2014, it was estimated that 1.5 million people died as a result of and 9.6 million fell ill with tuberculosis.^1^ Despite these alarming figures, efforts to reduce the annual tuberculosis incidence rate over the last decade have resulted in only a meagre 1.5% decline.^1^

In order to reach the ambitious targets of the Sustainable Development Goals by 2030 of reducing tuberculosis deaths by 90%, reducing the tuberculosis incidence rate by 90%, and ensuring that no tuberculosis-affected family is facing catastrophic costs due to tuberculosis, a paradigm shift is urgently needed.^1^ Recently, a series of papers was published in *The Lancet* on how to eliminate tuberculosis, suggesting repacking current interventions into a comprehensive control strategy.^2^ The World Health Organization End TB Strategy supports this and also emphasises the need for better adoption and rapid uptake of new tools to diagnose tuberculosis earlier, the systematic screening of high-risk populations, and the effective and rapid roll-out of these strategies in highly-affected countries.^3^ However, the practicality of achieving these components remains challenging because of the lack of a rapid, simple, accurate and affordable point-of-care diagnostic and screening algorithm that can be scaled-up to screen large numbers of individuals. Nevertheless, achievement of these goals is necessary and must catalyse the development of new interventions in Africa, for Africa, the continent with the highest tuberculosis mortality and morbidity rates.^1^

## Xpert^®^ MTB/RIF: the unfulfilled promise?

The most recent and widely-implemented new laboratory technology for the diagnosis of tuberculosis is the Xpert MTB/RIF^^®^^ (Cepheid, Sunnyvale, California, United States). Xpert MTB/RIF has been rolled-out in many resource-limited, high tuberculosis-incidence countries through the support of international donors. Although the Xpert MTB/RIF assay has proven to increase tuberculosis case detection,^4^ two recent randomised trials in Southern Africa suggest that introducing Xpert MTB/RIF alone may not significantly reduce tuberculosis-related morbidity and mortality,^5,6^ underscoring the fact that tuberculosis diagnostics do not function in isolation but are part of a cascade, which includes clinician assessment, treatment initiation decisions, and follow-up and adherence efforts.

In addition, an Xpert MTB/RIF instrument costs $17 000 and relies on testing cartridges that, even with concession pricing for developing countries, cost just under $10.00 per unit. The significant ongoing electricity needs, the variable costs and technological upkeep (maintenance and calibration) pose major barriers for the sustainability of this technology at the point of care in resource-limited settings.^7^ A full scale-up of Xpert MTB/RIF in Tanzania, for example, would require a 25% increase in the National Tuberculosis Programme’s budget and financial resources, which is unaffordable in the current fiscal climate.^8^

Although there is clearly a role for Xpert MTB/RIF in diagnosing tuberculosis and providing drug-susceptibility results in specific populations and in specific settings, without higher sample throughput and reduced costs, as well as robust linkages to care, the burden of tuberculosis will not drop. We agree with the Stop TB Partnership’s Global Plan to End TB 2016–2020 that a paradigm shift is needed, one that includes new and innovative programmes that can rapidly and efficiently roll out diagnostics, drugs and vaccines to impact the tuberculosis epidemic, especially in the developing world.^9^ However, to date most of the new laboratory tests under development in the diagnostic pipeline are molecular methods, which can be expensive and are unable to efficiently handle high sample throughput in decentralised settings.

The ideal tuberculosis diagnostic test would be low cost, patient- and user-friendly, accurate for all forms of tuberculosis in both HIV-negative and HIV-positive populations, produce immediate results at the point of care, have high throughput, and be well embedded and sustainable in developing countries within existing health care delivery structures.^10^ An example of this would be a urine-based, lateral-flow assay similar to the standard home pregnancy test. However, to our knowledge, there is nothing in the five-year tuberculosis diagnostic pipeline for review by the World Health Organization that meets all of these requirements.^11^

## An African solution

In the last decade, tuberculosis scent detection studies have been performed with animal, insect and electronic noses and may be a unique ‘outside-the-box’ solution to fill the tuberculosis diagnostic need in developing countries.^12^ Recently, research using specially-trained African giant pouched rats as detectors of pulmonary tuberculosis, has advanced to the stage where this technology is being used daily for tuberculosis screening for people living in Tanzania and Mozambique.^13^ Sputum samples are collected from presumptive tuberculosis patients who test Ziehl Neelsen smear-negative at public and private tuberculosis clinics in Dar es Salaam and Maputo. A team of detection rats subsequently re-screens these samples to identify tuberculosis through scent and diagnose tuberculosis cases that were missed by smear microscopy. Using a threshold of at least one detection rat indication to determine a positive result provides a sensitivity of 72%–80% and a specificity of 59%–74%, at an inexpensive cost per screening (Table 1).^14,15^ All the samples indicated positive by the rats currently undergo confirmation by concentrated light-emitting diode fluorescence microscopy (LED-FM). Confirmation with Xpert MTB/RIF is under consideration, given the higher sensitivity of Xpert MTB/RIF compared with LED-FM.

**TABLE 1:** Test characteristics of detection rats and tuberculosis diagnostics common in sub-Saharan Africa.

Assay	Sensitivity in HIV-negative patients		Specificity in HIV-negative patients		Sensitivity in PLHIV		DST	Cost per test (USD)		Turn-around time	Operational requirements
	%	No.	%	No.	%	No.		USD	No.		
Culture	100	19	100	19	100	-	Yes	Solid 12.35 Liquid 16.62	20	Solid up to 8 weeks Liquid up to 2 weeks	Biosafety level 3 laboratory
Smear microscopy (conventional ZN)	61	19	98	19	Sensitivity drops to 20	21	No	1.50	8	25–30 samples per day †	- Ventilation - Separate benches - Dedicated technician time
Digital CXR	90	11	80	11	Sensitivity drops to 80	11	No	6.90	8	40 patients per hour	- Adequate power supply - Trained radiologists - High upfront costs
Xpert MTB/RIF	89	4	99	4	Sensitivity drops to 61	4	Yes, Rifampicin	19.85	23	16 samples per day	- Climate control - Uninterrupted power supply - Calibration
Detection rats (using a threshold of 1 rat)	72	15	59	15	Independent of HIV status	15	No	0.92‡ (microcosting, in preparation)	-	100 samples per 20 minutes	- Adequate throughput of samples - Rat kennel - Rat caretaker

CXR, chest X-ray; DST, drug-susceptibility testing; PLHIV, people living with HIV; ZN, Ziehl Neelsen.

† Excluding confirmation with light-emitting diode fluorescence microscopy.

‡ UNITAID. TUBERCULOSIS Diagnostics Technology and Market Landscape 3RD EDITION. Geneva: 2014.

The detection rat technology is part of a larger community response where results are reported on the same day to a community-based organisation which is then required to track the tuberculosis-positive patients and ensure that they initiate anti- tuberculosis treatment. The overall advantage of rats is they can screen up to 100 samples in 20 minutes and can reduce workload by rapidly funnelling-down the number of smear ‘negative’ samples that require confirmatory testing to about 30% of the original number.^16^ Samples indicated positive by the rats but which are LED-FM negative are considered bacteriologically tuberculosis-negative but, at the discretion of the clinician, can: (1) undergo repeat tuberculosis testing; (2) be treated empirically for tuberculosis; (3) undergo further investigations/treatment for other conditions mimicking tuberculosis; or (4) simply be followed and asked to return to the clinic if symptoms do not resolve or worsen.

Using the tuberculosis detection rats in combination with LED-FM has significantly increased tuberculosis case detection in Tanzania since 2007 and in Mozambique since 2013. To date, there have been an additional 10 000 patients (a 40% increase) missed by the public health system that were diagnosed by the rats and confirmed by LED-FM.^17^ By providing same-day results, and with the support of community-based healthcare workers, a large number of patients can access tuberculosis treatment quickly, maximising individual clinical benefits and minimising community spread. Specific studies comparing the morbidity and mortality of rat detection compared to the standard of care have not yet been done, but need to be undertaken. Of relevance to Africa, the sensitivity of rats does not seem to be affected by the HIV status of the patient^15^ (unlike smears, which have been found to have sensitivities as low as 26% in new HIV clinic enrolees^18^).

Detection rat technology has been rigorously tested and researched for over 10 years. The trained rats target a blend of specific volatile organic compounds produced by *Mycobacterium tuberculosis*.^24^ The rat detects tuberculosis by walking through a rectilinear cage with holes in the floor where small pots with different patient’s sputum specimens are placed, each covered with a sliding lid. As the rat approaches a pot, the technician slides the lid open for the rat to sniff the sputum. In the proof-of-principle study, Weetjens et al. showed that rats could be trained by operant conditioning (positive reinforcement) to pause for at least five seconds at holes where a sputum sample was positive for tuberculosis (as confirmed by solid culture), but not to pause at holes where the sputum sample was tuberculosis-negative.^25^ These research results were sufficiently promising that in 2007 the Tanzanian Ministry of Health allowed the rats to be used for second-line screening of sputum samples from presumptive tuberculosis patients who presented at tuberculosis clinics.^25^ The Mozambique Ministry of Health followed suit in 2013. Other studies have shown that teams of rats, which are fast and affordable, improve case detection well above that of directly-observed treatment, short-course clinic microscopy.^13,14,26^

In Tanzania, there is a functioning breeding programme run by a Belgian non-governmental organisation – APOPO (‘Anti-Persoonsmijnen Ontmijnende Product Ontwikkeling’; www.apopo.org) – in collaboration with the Sokoine University of Agriculture which supplies rats ‘as needed’ for the training, research and detection programmes currently underway. Training tuberculosis detection rats takes an average of nine months, but they have a working lifespan of up to seven years. Training one rat costs about $1000 and the cost of having one sample evaluated by a team of four rats is around $0.92, assuming a throughput of 1000 samples a week, which is the current throughput for 24 clinics in Dar es Salaam (manuscript in preparation). The cost per sample screened could potentially drop to around $0.70 through scale-up as more samples are screened. While there are some variable costs associated with screening more samples by rats, they are far less than the costs associated with screening extra samples with, for example, Xpert MTB/RIF. This is because each Xpert MTB/RIF examination requires a standard cost for each cartridge used.^23^ The unlimited access to the rats from the region, combined with an intensive rat trainer accreditation programme, also run by APOPO, that includes mentoring new handlers, helps to ensure that APOPO’s work is sustainable and the impact is scalable. Rat performance and accuracy is monitored daily by an intensive quality assurance programme. Known positive and known negative samples are placed randomly for the rats to examine and individual rat performance is assessed.

Because of the rapid diagnostic speed and low variable costs of detection rats, this technology is particularly well suited to screening the large numbers of patients found in African cities where tuberculosis is concentrated in heavily-populated, informal settlements characterised by substandard housing and low income. In these settings, public health laboratories are typically unable to maintain the quality of smear microscopy due to the high throughput of tuberculosis samples, resulting in many false-negative results. Using an efficient motorcycle sputum transportation system, it is possible to have all samples shipped to one quality-controlled central laboratory to undergo screening by detection rats and confirmation by LED-FM, with a turn-around time of one day.

Large, congested metropolitan centres not only act as a focus of local tuberculosis infection, but may also facilitate the spread of tuberculosis infection countrywide due to the extensive migration in and out of the cities. Innovative and affordable approaches, such as tuberculosis detection rats that offer high sample throughput, could be a solution to successfully tackle tuberculosis burden in these areas and would be in line with the Zero TB Cities Project which focuses on finding the ‘how’ for tackling infectious diseases with local tools in local contexts, while not lowering standards (http://www.advanceaccessanddelivery.org/). Detection rat algorithms may also be used as a cost-effective, active case-finding tool in key high-risk populations, such as correctional facility inmates, miners and refugees. Whether increases in case detection through the use of detection rats has an impact on reducing tuberculosis morbidity and mortality and whether this is cost-effective needs further study.

### Conclusion

In order to accelerate the elimination of tuberculosis in sub-Saharan Africa, a multi-pronged approach is required. Until new, sustainable and inexpensive laboratory tuberculosis diagnostic tests are discovered, commercialised, validated and scaled up, other innovative diagnostic solutions, such as tuberculosis detection-trained African pouched rats, must be used in combination and in collaboration with tuberculosis laboratories in Africa. This will require countries and international donors to adopt and roll-out complementary technologies for the various settings where they work best. Rat scent detection technology, working in concert with laboratories, provides an opportunity to play a major complementary role in improving tuberculosis detection rates in low resource, high tuberculosis-burden urban settings for the foreseeable future.
